# Preoperative evaluation of pulmonary artery morphology and pulmonary circulation in neonates with pulmonary atresia - usefulness of MR angiography in clinical routine

**DOI:** 10.1186/1532-429X-12-52

**Published:** 2010-09-15

**Authors:** Nadine Kawel, Emanuela Valsangiacomo-Buechel, Ricarda Hoop, Christian J Kellenberger

**Affiliations:** 1 Department of Diagnostic Imaging, University Children's Hospital Zurich, Switzerland; 2 Department of Radiology, University Hospital Basel, Switzerland; 3 Division of Paediatric Cardiology, University Children's Hospital Zurich, Switzerland

## Abstract

**Background:**

To explore the role of contrast-enhanced magnetic resonance angiography (CE-MRA) in clinical routine for evaluating neonates with pulmonary atresia (PA) and to describe their pulmonary artery morphology and blood supply.

CE-MRA studies of 15 neonates with PA (12 female; median weight: 2900 g) were retrospectively evaluated by two radiologists in consensus. Each study was judged to be either diagnostic or non-diagnostic depending on the potential to evaluate pulmonary artery morphology and pulmonary blood supply. In those cases where surgery or conventional angiocardiography was performed results were compared.

**Results:**

CE-MRA was considered diagnostic in 87%. Pulmonary artery morphology was classified as "confluent with (n = 1) and without (n = 1) main pulmonary artery", "non-confluent" (n = 6) or "absent" (n = 7). Source of pulmonary blood supply was "a persistent arterial duct" (n = 12), "a direct" (n = 22) or "indirect (n = 9) aortopulmonary collateral artery (APCA)" or "an APCA from the ascending aorta" (n = 2). In no patient were there any additional findings at surgery or conventional angiocardiography which would have changed the therapeutic or surgical approach.

**Conclusions:**

CE-MRA is a useful diagnostic tool for the preoperative evaluation of the morphology of pulmonary arteries and blood supply in neonates with PA. In most cases diagnostic cardiac catheterization can be avoided.

## Background

Pulmonary atresia (PA) is classified into pulmonary atresia with ventricular septal defect (PA-VSD) and PA with intact ventricular septum (PA-IVS). PA-VSD may occur with any form of congenital heart disease (CHD) like tetralogy of Fallot (TOF), double outlet right ventricle, double outlet left ventricle and transposition of great arteries [1] while PA-IVS is an uncommon form of congenitally malformed heart [2].

The site of atresia varies and any part of the right ventricular outflow tract - the infundibulum, the pulmonary valve and the main pulmonary artery - may be atretic. The main pulmonary artery may be completely absent while the right and left pulmonary arteries may be hypoplastic, either confluent or non-confluent, or absent [3]. Knowledge of the exact morphology of the pulmonary arteries as well as the presence and course of aortopulmonary collateral arteries (APCAs) or patent ductus arteriosus (PDA) is crucial for the treatment strategy of these patients and for concrete planning of the surgical or catheter-guided interventions required [1,4,5].

Cardiovascular magnetic resonance (CMR) is regarded as a useful tool for diagnosis and preoperative evaluation of CHD as it is less invasive than conventional angiocardiography and does not involve ionizing radiation [6-9]. Contrast-enhanced magnetic resonance angiography (CE-MRA) is considered helpful in the evaluation of pulmonary artery morphology [7,10] and of major APCAs [11,12] in older children and adults with TOF or PA.

Our aim was to explore the role of CE-MRA in clinical routine for evaluating neonates with pulmonary atresia and to describe their pulmonary artery morphology and blood supply.

## Methods

This retrospective study was conducted at a cardiology centre of a tertiary paediatric hospital and was approved by the institutional research ethics board. Children with PA who underwent CE-MRA during the neonatal period for initial workup were identified, and their clinical charts and imaging studies were reviewed.

### Patient population

Search of the electronic databases of the departments of cardiology and radiology, including the period from January 2002 to October 2008, revealed 40 neonates with PA (26 PA-VSD, 14 PA-IVS) referred to the hospital during the first 28 days of life. Of the 26 patients with PA-VSD, 14 had CE-MRA during the neonatal period, whereas 2 underwent CE-MRA after the neonatal period. 10 of the 26 neonates with PA-VSD did not undergo CE-MRA preoperatively: one patient died during the first days of life, 2 underwent conventional angiocardiography at cardiac catheterization for interventional purposes, while during the introduction phase (2002-2004) of CE-MRA at our institution 7 patients had cardiac catheterization exclusively for diagnostic purposes. Of 14 patients with PA-IVS, only 1 had a preoperative CE-MRA, while 3 died within the first days of life without any angiography, 8 had diagnostic conventional angiography at catheterization for interventional purposes, and 2 had diagnostic catheterization solely to evaluate communications between the right ventricle and the coronary arteries.

Thus, the study population included 15 patients (12 female and 3 male) with PA (14 PA-VSD, 1 PA-IVS) who underwent CE-MRA for initial diagnosis at a median age of 3 days (range: 1-27 days) with a median weight of 2900 g (range: 1500-3900 g). The main cardiac anomalies are listed in table 1. Additional cardiac defects consisted of total anomalous pulmonary venous connection in three patients, transposition of the great arteries in 5 patients, and hypoplastic left ventricle in two patients. Extracardiac anomalies occurred in 5 patients, including nonspecific dysmorphic syndrome, Allagile syndrome, anal atresia, microcephaly, dandy walker malformation, hypoplasia of the corpus callosum and hypoxic ischemic encephalopathy.

**Table 1:** Main cardiac anomaly in 15 neonates with PA

Main cardiac anomaly	Number of patients
Isolated PA-VSD	8
Isolated PA-IVS	1
Complex PA-VSD:	
PA and Tricuspid atresia	1
Heterotaxy syndrome and atrioventricular septal defect (AVSD)	3
Atrioventricular and ventriculoarterial discordance (L-TGA)	2

14 patients underwent surgery and/or a catheter guided intervention between 3 and 324 days (median: 28 days) following the initial CMR study, while 1 patient died prior to an intervention. In 3 patients total repair with a single stage unifocalization and reconstruction of the right ventricular outflow tract was performed. 8 patients received a modified Blalock-Taussig or central aortopulmonary shunt with concomitant reconstruction of the pulmonary arteries and unifocalization of APCAs in 6 cases. Unsuccessful stenting of bilateral PDAs in one patient and of a stenotic APCA in another patient was followed by surgery with placement of a modified Blalock-Taussig shunt and reanastomosis of the APCA to the aorta. The patient with PA-IVS underwent a Rashkind atrioseptostomy but died before surgery. Total anomalous pulmonary venous connections were repaired at the initial surgery in 2 of 3 patients.

### Imaging technique

The CMR studies were performed on a 1.5-T scanner (Signa Twinspeed, GE Medical Systems, Milwaukee, Wisconsin) using a quadrature head coil. Coronal, axial and sagittal nongated steady-state free precession (SSFP) images covering the entire chest served as localizers. For CE-MRA, three-dimensional fast spoiled gradient echo (3D FSPGR) data sets were acquired with coronal partitions and following technical parameters: flip angle 30°; mean echo time 1.17 ms (range: 1.00-1.43 ms), mean repetition time 3.42 ms (range: 3.31-3.68 ms), mean section thickness 1.9 mm (range: 1.6-2.8 mm), mean field of view 216 mm (range: 200-290 mm), matrix 256 × 160. With zero fill interpolation in all three dimensions (ZIP 4, ZIP 512) the spatial resolution was reconstructed to 0.48 × 0.42 × 0.42 mm^3 ^voxel size on average. A double dose (0.2 mmol/kg body weight) of gadolinium-based contrast material (dimeglumine gadopentate, Magnevist^®^, Bayer AG, Switzerland; or gadodiamide, Omniscan^®^, GE Healthcare AG, Switzerland) was injected manually through a peripheral intravenous line over approximately 5 seconds, followed by an equivalent volume of saline solution. For optimal timing an automated bolus detection method (Smartprep, GE Medical Systems) was used in 7 patients, while in 8 patients the scan was started manually after a delay of 4 to 6 seconds. All studies were performed under general anaesthesia with intubation allowing three sequential data acquisitions during a single breath hold of 45 - 60 seconds.

Depending on the underlying congenital heart disease or the associated cardiac anomalies in some cases CMR also included cine imaging with electrocardiographically gated fast gradient-echo sequences and velocity-encoded phase-contrast cine sequences.

### Image evaluation

The CE-MRA was evaluated retrospectively by two radiologists in consensus, who were blinded to the results of other imaging studies and findings at surgery. The original image data were viewed on a workstation (Advantage Windows version 4.2, GE Medical Systems) using multi-planar reconstruction (MPR), maximum-intensity-projection (MIP) and volume rendering (VR) techniques. Each CMR study was judged to be either diagnostic or non-diagnostic depending on the potential to definitively affirm or negate the presence of native pulmonary arteries and to describe and classify pulmonary artery morphology and the source of pulmonary blood supply according to the classification mentioned below. If pulmonary arteries or pulmonary blood supply could not be completely evaluated and additional cardiac catheterization had to be recommended, the study was judged non-diagnostic. Findings of the consensus reading were compared to those of the initial CMR report, conventional angiocardiography report and cine documentation (n = 5), and to the description of the anatomy in the surgical report (n = 13). Comparison to angiocardiographic or surgical findings was available in all but one patient.

A pulmonary artery was defined as a vessel entering the lung at the hilum with a typical branching accompanying the bronchial tree. According to Tchervenkov et al. [1] PA was defined as "the lack of luminal continuity and absence of blood flow from a ventricle or a rudimentary chamber and the pulmonary artery". Pulmonary artery morphology was classified into the categories "confluent pulmonary arteries with main pulmonary artery", "confluent pulmonary arteries without main pulmonary artery", "non-confluent pulmonary arteries" and "complete absence of native pulmonary arteries" [3]. In case of non-confluent pulmonary arteries the distance between the right and left pulmonary artery was measured. The source of pulmonary blood supply was classified into "patent arterial duct" (arising from the undersurface of the aortic arch or the innominate artery or proximal subclavian artery on the opposite side of the aortic arch), "direct aortopulmonary collateral artery" (arising usually from the descending thoracic aorta and rarely from the aortic arch or the abdominal aorta), "indirect aortopulmonary collateral artery" (arising from arterial branches of the aorta) and "aortopulmonary collateral artery from the ascending aorta". An arterial duct should be a single source of blood supply to both lungs or one lung when the pulmonary arteries are not confluent (reciprocal rule for APCA and PDA) [1-3,13].

For each vessel origin, destination and diameter were documented as well as the presence and localisation of a stenosis of more than 50% of the luminal diameter.

## Results

**Figure 1:**
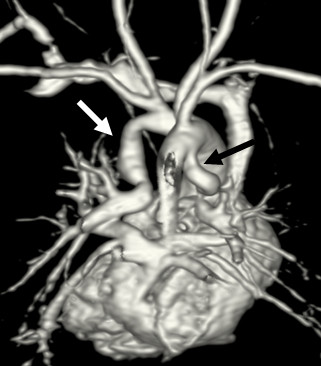
**7-day-old girl with complex cardiac malformation including right isomerism, transposition of the great arteries and pulmonary atresia with non-confluent pulmonary arteries**. 3D volume rendered CE-MRA image from posterior (with partial removal of the descending aorta) shows blood supply to the right lung via a PDA (*black arrow*) originating from the undersurface of the aortic arch, anastomosing with the native pulmonary artery at the hilum and with a distal stenosis > 50%. Blood supply to the left lung via a PDA (*white arrow*) originating from the innominate artery and anastomosing with the native pulmonary artery at the hilum.

**Figure 2:**
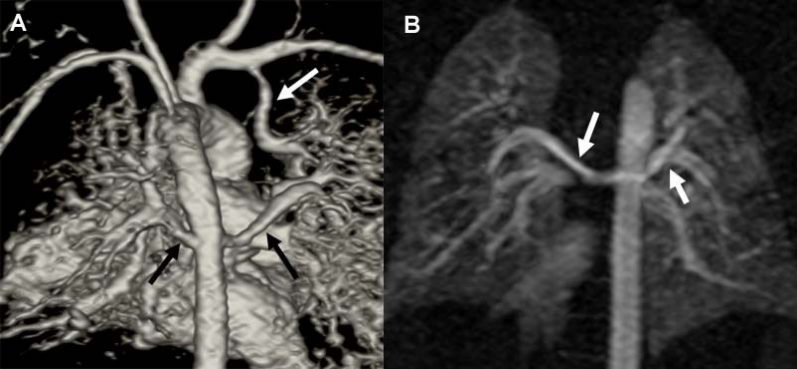
**a, b 3-day-old boy with pulmonary atresia and complete absence of native pulmonary arteries**. 3D volume rendered image from posterior (**a**) and coronal subvolume MIP image viewed from anterior (**b**) show blood supply to right and left lungs via direct APCAs originating from the descending aorta (**a**, *black arrows*; **b**, *white arrows*) and an indirect APCA (**a**, *white arrow*) originating from the right subclavian artery supplying the right upper lobe.

**Figure 3:**
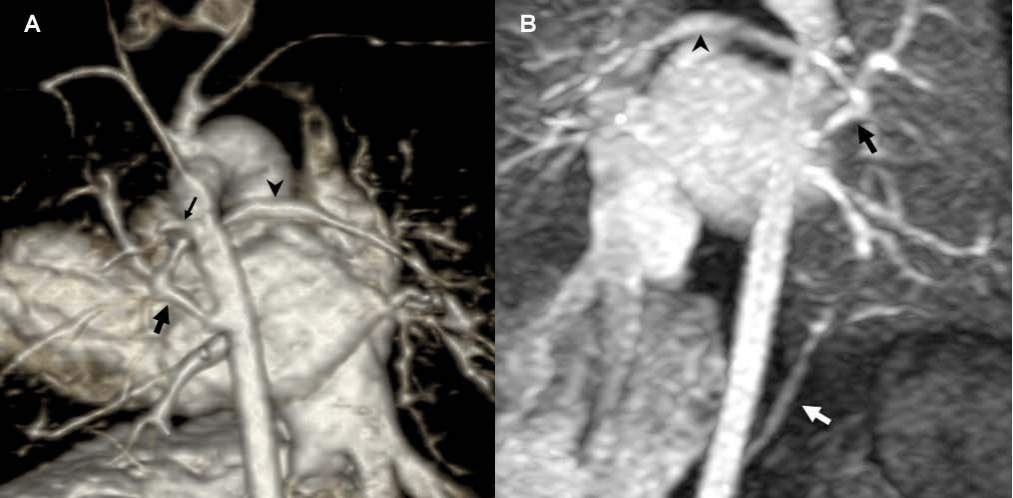
**a, b 1-day-old girl with pulmonary atresia**. 3D volume rendered image from posterior (**a**) and coronal-oblique MIP image viewed from anterior (**b**) show blood supply to both lungs via direct APCA originating from the descending aorta (**a **and **b**, *bold black arrow*), which anastomoses with the left pulmonary artery at the hilum and supplies confluent hypoplastic pulmonary arteries without a main pulmonary artery (**a **and **b**, *arrowhead*). Blood supply to the left upper lobe derives from another direct APCA (**a**, *thin black arrow*) originating from the descending aorta. The left lower lobe is supplied via an indirect APCA deriving from the celiac trunk (**b**, *white arrow*).

All 15 neonates with PA underwent CMR without complication. The pulmonary vasculature was best delineated on the first acquisition of the CE-MRA in 87%, whereas the second set of images showed the pulmonary arterial anatomy more clearly in 2 cases (one case each with automatic bolus detection and best-guess methods for detection of contrast arrival). In all cases the pulmonary artery morphology (table 2) could be categorized and the source of pulmonary blood supply identified. In all 6 patients with non-confluent pulmonary arteries, bilateral PDAs extending from the undersurface of the aortic arch or the innominate artery to the native pulmonary arteries at the hila were the sole pulmonary blood supply (Fig. 1). The distance between the non-confluent pulmonary arteries measured between 1 and 15 mm (mean: 7 mm). In the 2 patients with confluent pulmonary arteries (Fig. 2) and the 7 patients with complete absence of the native pulmonary arteries (Fig. 3), APCAs were the source of pulmonary blood supply. Overall 33 APCAs were identified (2-6 APCAs per patient) with a stenosis of more than 50% of the lumen in 8 vessels. Diameters of the APCAs varied between < 1 mm and 4.5 mm. Origins and destinations of APCAs are shown in table 3. The destination of APCAs could be defined only to the level of the supplied lung lobe(s) but not assigned to lung segments. Potential intrapulmonary connections between APCAs and native pulmonary arteries could not be identified. Despite these shortcomings, 13 of the 15 CE-MRAs were considered diagnostic providing all morphologic details required for deciding on treatment and planning surgery. However, during the introduction phase of CE-MRA at our institution, 1 patient underwent additional diagnostic catheterization for confirming the absence of native pulmonary arteries. Another 3 patients had catheterization for interventional purposes (Rashkind balloon atrial septostomy, stenting of an APCA, and stenting of bilateral PDAs).

**Table 2:** Morphology of pulmonary arteries in 15 neonates with PA

Pulmonary artery morphology	Number of patients
confluent pulmonary arteries with main pulmonary artery	1
confluent pulmonary arteries without main pulmonary artery	1
non-confluent pulmonary arteries	6
complete absence of native pulmonary arteries	7

**Table 3:** Aortopulmonary collateral arteries in 15 neonates with PA

APCA	origin	destination	n**
direct	descending aorta	whole right or left lung	7
		
		upper lobe	5
		
		lower lobe and middle lobe	2
		
		lower lobe	3
		
		pulmonary artery *	2
		
		lower lobe and lingula	1
	
	aortic arch	lower lobe and middle lobe	1
		
		lower lobe	1

indirect	subclavian artery	right and left lung via confluent pulmonary arteries *	1
		
		upper lobe	5
	
	left coronary artery	lower lobe	1
	
	coeliac trunc	lower lobe	2

from the ascending aorta		lower lobe	1
		
		lower lobe and lingula	1

* anastomosis in the region of the hilum, ** n = number of APCAs

In none of the 9 patients who underwent surgery only with CE-MRA were there any additional intraoperative findings that would have changed the therapeutic strategy or surgical approach. Nonetheless, there were discrepant findings between CE-MRA, surgery and conventional angiocardiography in 3 cases. In the first patient, an APCA arising from the left coronary artery was misinterpreted as a hypoplastic native pulmonary artery in the initial CMR report and the consensus reading. In knowledge of the surgical report, this vessel could be identified on CE-MRA as indirect APCA arising from the left coronary artery and supplying the right lower lobe. In the second patient, a direct APCA from the descending aorta branching into the right lower lobe was not described at surgery performed 8 weeks after the CMR study but was confirmed by angiocardiography 10 weeks later. In the third patient, an APCA arising from the left subclavian artery supplying the left upper lobe with a diameter of less than 1 mm was diagnosed only at cardiac catheterization for a Rashkind balloon atrial septostomy, although it could be identified retrospectively on CE-MRA.

In 2 cases CE-MRA was judged as non-diagnostic because direct APCAs from the descending aorta could only be suspected but not clearly identified due to intensely enhancing surrounding atelectatic lung in one case and a vessel diameter of less than 1 mm in the other case. Catheter angiocardiography demonstrated the direct APCAs and confirmed the remainder of the pulmonary artery morphology in the first case, while in the second case the planned catheterization was not performed because the patient died of a gastrointestinal infection. Overall, 5 of the 15 patients underwent additional catheterization following CE-MRA, 2 for diagnostic and 3 for interventional purposes.

## Discussion

The anatomy and morphology of the pulmonary circulation determines treatment planning and surgical approach in patients with PA [1]. Traditionally, cardiac catheterization has been performed for preoperative angiographic evaluation of PA [7] with a considerable complication rate and radiation exposure [14,15]. CMR is regarded as a less invasive option and is recently being increasingly used in the assessment of CHD [6-9]. With CE-MRA the pulmonary arteries [7,10] and APCAs [11,12,16] can be well evaluated. By comparing CE-MRA and cardiac catheterization in 32 patients (age range of 1 day to 46.9 years; median 4.7 years) with complex PA, Geva et al. [11] considered CE-MRA an accurate alternative to diagnostic cardiac catheterization for the delineation of all sources of pulmonary blood supply. Although a few neonates were included in their comparison, there is general believe that CE-MRA may not suffice in the delineation of the small pulmonary vessels in neonates. While Roche et al. [16] were able to describe all major APCAs (≥ 5 mm) by combining cine gradient-recalled echo imaging and CE-MRA detected by conventional angiography in 11 patients (mean age 9 years), they found CMR less effective in the detection of minor APCAs (< 5 mm) and determination of the supplied lung.

Our study demonstrates that CE-MRA is very well suited to depict small pulmonary vessels when the spatial resolution is optimized. We were able to correctly describe the presence and morphology or absence of native pulmonary arteries in 93% (14/15 patients) of the investigated neonates. Non-confluent pulmonary arteries were supplied by bilateral PDAs in all cases, which has been considered a rare condition by some [17] but also recognized as quite common by others [18]. In a study by Harikrishnan et al. pulmonary blood supply was provided by a PDA in 49 of 86 patients with tetralogy of Fallot and PA [19]. In two patients APCAs originating from the ascending aorta were found, which to our knowledge has not been mentioned in any other CMR study, but could represent a persisting fifth aortic arch as described by Freedom [2]. All APCAs seen in our neonates had a maximum diameter of 4.5 mm. Imaging in a head coil during breathhold with adaption of the field of view and slice thickness to the size of the baby provided sufficient spatial resolution to delineate origin, course and destination of APCAs as small as 1 mm diameter. The spatial resolution was not sufficient for delineation of intrapulmonary connections between APCAs and native pulmonary arteries, and the course of the APCAs could only be traced to the level of the supplied lung lobes but not to the level of the supplied lung segments. Despite these limitations, CE-MRA was considered "diagnostic" in 87% (13 of 15 patients) providing all morphologic details required for deciding on treatment and operative planning thus avoiding catheterization for diagnostic purposes.

The main limitation of this study is the lack of direct comparison between CE-MRA and conventional angiography in all cases. In our institution, since its introduction, CE-MRA has gradually replaced cardiac catheterization for the initial evaluation of the pulmonary vasculature in neonates and today it is the primary modality for evaluating patients with PA as long as no immediate intervention is required. Thus performing an invasive procedure such as additional cardiac catheterization for study purposes would have been unethical. The impact of changing the diagnostic algorithm, from cardiac catheterization to CMR, on the outcome of neonates with PA still needs to be demonstrated. Survival and quality of life of these children is primarily determined by the native anatomical conditions of the disease and by the ability of the surgeon in reconstructing a viable pulmonary arterial tree [20,21]. Nevertheless, we believe that restricting invasive catheterization to catheter-guided interventions may contribute to lower morbidity by reducing catheter-related thrombosis and potential tumour induction from high cumulative doses of radiation. This may be particularly important in this CHD that often requires repeated diagnostic and therapeutic procedures before achieving total repair.

Besides depiction of the pulmonary arterial anatomy, CMR may also provide haemodynamic information by obtaining additional velocity-encoded phase-contrast cine sequences. In patients with PA, flow volume measurements in the ascending aorta and in each pulmonary vein can be used to estimate the pulmonary-to-systemic blood flow ratio (QP/QS = QPV/(QAO-QPV)) and the amount of blood flow to each lung. The contribution of a PDA or large APCA to the pulmonary blood supply may be assessed, but investigation of all APCAs would be tedious and imprecise if they are small. With optimisation of current CMR techniques for imaging neonates, it should be possible to measure flow volumes with sufficient accuracy in vessels larger than 2 mm diameter [22,23].

## Conclusions

In conclusion, CE-MRA is a useful diagnostic tool in clinical routine for the preoperative evaluation of the morphology of pulmonary arteries and pulmonary circulation in neonates with PA. In most cases additional diagnostic cardiac catheterization can be avoided.

## Competing interests

The authors declare that they have no competing interests.

## Authors' contributions

NK, RH, EV and CK were involved in the study concept and design and performed data acquisition. NK and CK were involved in data analysis/interpretation. NK, EV and CK were involved in either manuscript preparation or editing. All authors read and approved the final manuscript.
